# From suspected lymphoma to self-limiting lymphadenitis: Kikuchi–Fujimoto disease in focus

**DOI:** 10.1007/s12308-026-00709-2

**Published:** 2026-06-03

**Authors:** Julian Rieck, Carsten Dittmayer, Martin Janz, David Horst, Simon Schallenberg

**Affiliations:** 1 Institute of Pathology, Charité – Universitätsmedizin Berlin, Berlin, Germany; 2 Hematology, Oncology and Cancer Immunology, Charité - Universitätsmedizin Berlin, Berlin, Germany; 3 Biology of Malignant Lymphomas, Max Delbrück Center for Molecular Medicine, Berlin, Germany; 4 Experimental and Clinical Research Center (ECRC), Charité and Max Delbrück Center for Molecular Medicine, Berlin, Germany

**Keywords:** Kikuchi–Fujimoto disease, Necrotizing lymphadenitis, Lymphoma mimics, Crescent-shaped histiocytes, Plasmacytoid dendritic cells, Nuclear dust

## Abstract

Kikuchi–Fujimoto disease (KFD) is a rare, self-limiting form of necrotizing lymphadenitis that primarily affects young adults and often mimics lymphoma or autoimmune lymphadenitis both clinically and histologically. We report the case of a 28-year-old Caucasian woman with a history of Hashimoto’s thyroiditis presenting with progressive cervical lymphadenopathy, fever, weight loss, and myalgia. Laboratory findings showed leukopenia, elevated transaminases, and increased LDH. Imaging revealed bilateral lymphadenopathy and mildly enlarged spleen. Histopathological examination of an excised lymph node demonstrated necrotizing lymphadenitis with crescent-shaped histiocytes, plasmacytoid dendritic cells, and karyorrhectic debris. Immunohistochemistry supported the diagnosis and excluded lymphoma, while electron microscopy did not provide evidence of infection. The patient recovered fully without specific treatment. This case highlights the importance of recognizing the characteristic features of KFD to avoid misdiagnosis and overtreatment. Given its potential association with autoimmune disease and recurrence, clinical follow-up is recommended.

## Introduction

Kikuchi–Fujimoto disease, first described in 1972 by Japanese pathologists Kikuchi [1] and Fujimoto [2], is a rare, self-limiting lymphadenitis predominantly affecting young adults. Despite being a benign condition, KFD often mimics lymphoma clinically and histologically [3–5], presenting a significant diagnostic challenge. The etiology of KFD remains elusive, with hypotheses ranging from infectious triggers to autoimmune mechanisms. Agents of viral, bacterial, and protozoan origin have been discussed, though definitive evidence is lacking [6–9].

Associations with autoimmune diseases are increasingly recognized [10, 11], with shared histological features complicating differentiation [12]. Associated autoimmune conditions include systemic lupus erythematodes, Hashimoto’s disease [13, 14], Sjögren’s disease [15], and systemic sclerosis [16], as well as multiple sclerosis [17] and limbic encephalitis [18].

Clinically, KFD presents as acute to subacute cervical lymphadenopathy accompanied by fever [19], malaise, weight loss, arthralgias, hepatomegaly, splenomegaly, and various skin manifestations [20, 21]. Possible changes in blood samples include leukopenia [19, 22] and an elevated erythrocyte sedimentation rate [23].

Histopathologically, KFD is characterized by partial or complete lymph node involvement with necrosis containing karyorrhectic debris (nuclear dust), crescent-shaped histiocytes, and plasmacytoid dendritic cells [24].

The necrotic areas are typically devoid of neutrophils and typically lack plasma cells [20]; however, overt necrosis is not a prerequisite for diagnosis [25]. Surrounding these pale necrotic regions an abundance of small lymphocytes can be observed [26].

Immunohistochemically, KFD lesions show a T-cell-rich profile with strong CD3 and CD4 or CD8 positivity, the latter being predominant [26]. Crescent-shaped histiocytes may stain positive for myeloperoxidase [27] and lysozyme [3, 28], while showing weak CD4 expression.

Plasmacytoid dendritic cells also stain weakly for CD4 but exhibit a strong positivity for CD123 and TCL1 [29]. Notably, karyorrhectic areas lack CD20-positive B cells [26].

Ultrastructural features of KDF have been described, with intracytoplasmic myelin-like inclusions in immunoblasts and histiocytoid cells [30].

Although advances have been made in identifying genetic associations with KFD, no specific molecular markers currently exist to definitively confirm the diagnosis [31]. The exclusion of infectious causes of lymphadenopathy, particularly viral agents not associated with KFD may aid in differential diagnosis [6, 8, 9, 32, 33].

The standard management of KFD primarily involves supportive care and observation [9, 34]. In severe cases, glucocorticoids may be required [10, 35], and some patients have shown benefit from hydroxychloroquine [7, 10]. In refractory or recurrent cases, interleukin-1-receptor-inhibitors, such as anakinra, have demonstrated promising results [36]. KFD is generally a benign, self-limiting condition, with most cases resolving within 1–6 months [20, 23, 37, 38]. However, rare fatal outcomes have been documented [39–41].

Recurrence rates range from 3 to 21% [9, 10, 23, 42]. Histologically, KFD shares overlapping features (Table 1) with autoimmune-associated lymphadenitis [11, 22, 23], particularly systemic lupus erythematosus [8, 12]. Clinically and morphologically, it can closely mimic lymphoma [3, 5]. Diagnosis relies on lymph node biopsy and recognition of the characteristic histopathologic findings [7].

**Table 1:** Histologic features of Kikuchi–Fujimoto disease, systemic lupus erythematodes and lymphoma

Feature	Kikuchi–Fujimoto disease	Systemic lupus erythematodes	Lymphoma
Architecture	Irregularly shaped pale areas	Follicular and paracortical hyperplasia	Diffuse infiltrate of medium to large cells
Necrosis	Common; center of lesions	Common; center of lesions	Common
Neutrophils	Absent	Common	Common
Lymphocytes	Nonclonal population; CD8 + > CD4 +	Nonclonal population; CD8 + < CD4 +	Clonal, monotonous or atypical population
Special cells	Crescentic histiocytes; plasmacytoid dendritic cells	Plasma cells with Russell bodies	Reed-Sternberg cells (i.e.)
Extracellular	Nuclear dust	Hematoxylin bodies	Nuclear dust; amyloid

This case report describes a young female with suspected lymphoma who was ultimately diagnosed with KFD. This case drew our attention because clinical presentation and laboratory findings were highly suggestive of lymphoma, yet histopathology revealed KFD as the underlying cause.

## Case presentation

**Fig. 1:**
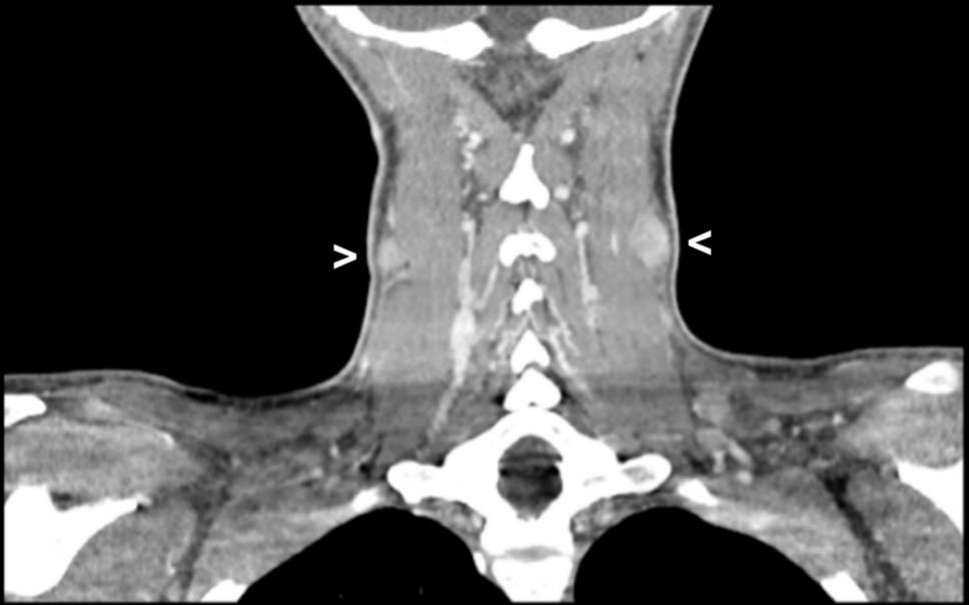
Radiologic presentation of cervical lymphadenopathy. Computed tomography scan of the neck showing bilateral cervical lymphadenopathy (arrowheads)

A 28-year-old Caucasian female with a known history of Hashimoto’s thyroiditis presented with progressive cervical lymphadenopathy persisting for 6 weeks. Accompanying symptoms included fever (38.5 °C), night sweats, a 7-kg weight loss, and generalized myalgia. Laboratory evaluation revealed mild leukopenia, lymphopenia, elevated transaminases, and an increased level of lactate dehydrogenase (LDH). Serologic testing for HIV, Epstein-Barr virus (EBV), and *Toxoplasma gondii* was negative. A computed tomography (CT) scan demonstrated bilateral cervical lymphadenopathy (Fig. 1) and mildly enlarged spleen. Excisional biopsy of a cervical lymph node was performed for histological evaluation.

**Fig. 2:**
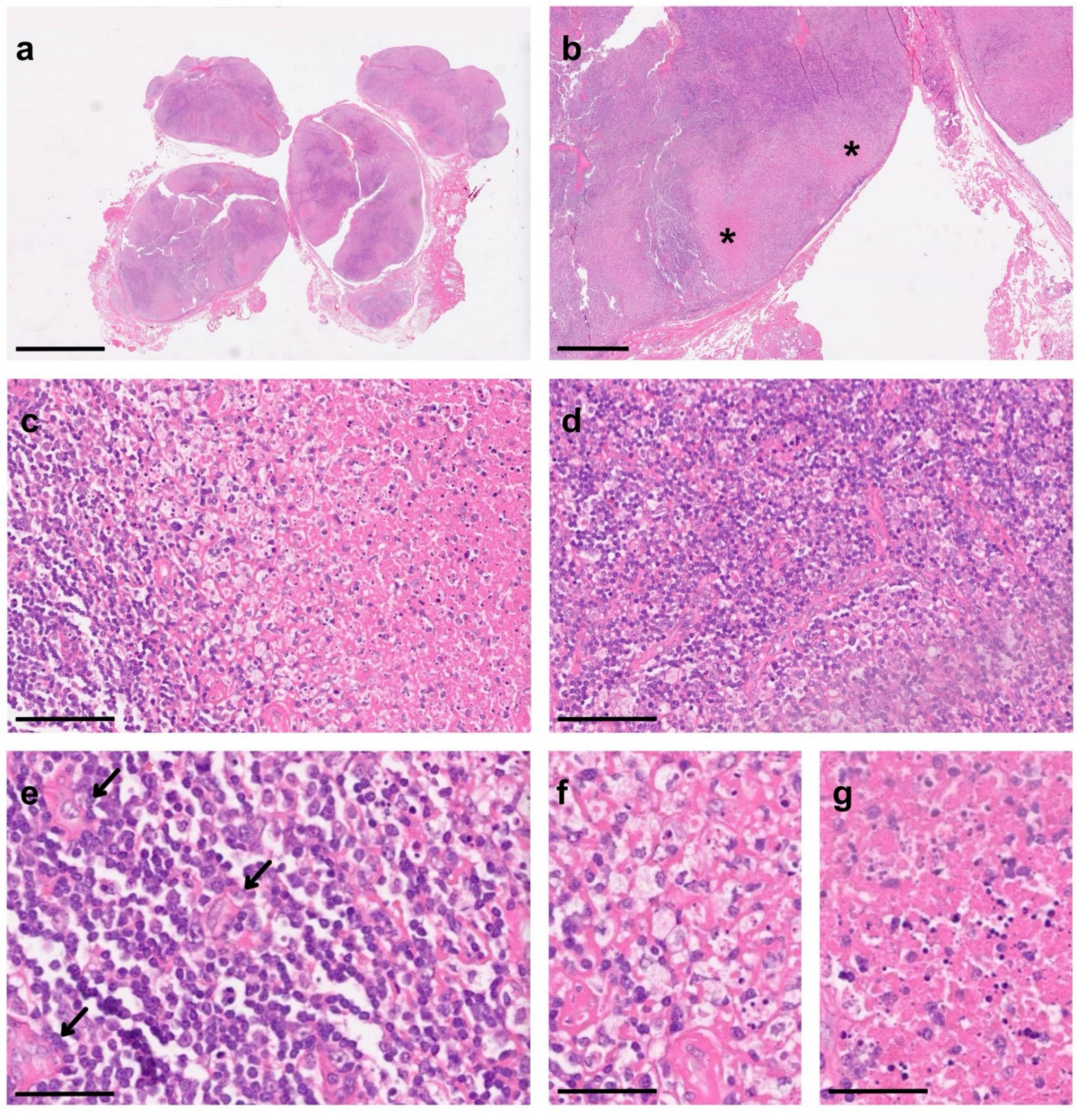
Hematoxylin and eosin (H&E) staining of the excised lymph node. **a** Lymph node with disrupted architecture and pale necrotic areas. **b** Higher magnification of subcapsular necrosis (asterisks). **c** Interface zone with apoptotic cells and small lymphocytes. **d** Abundant medium-sized lymphocytes adjacent to necrosis. **e** Crescent-shaped histiocytes interspersed among lymphocytes (arrows). **f** Foamy macrophages within necrotic areas. **g** Karyorrhectic debris (“nuclear dust”) within necrosis. Scale bars: **a** = 5 mm; **b** = 1 mm; **c**, **d** = 100 µm; **e**–**g** = 50 µm

Hematoxylin and eosin (H&E), PAS, and Giemsa stains revealed a lymph node with a thin, intact capsule and markedly disturbed architecture (Fig. 2a). Extensive subcapsular necrosis was present (Fig. 2b), containing scattered apoptotic cells (Fig. 2c), medium-sized lymphocytes (Fig. 2 d and e), crescent-shaped histiocytes (Fig. 2e), numerous foamy macrophages (Fig. 2f), and abundant nuclear debris (Fig. 2g).

**Fig. 3:**
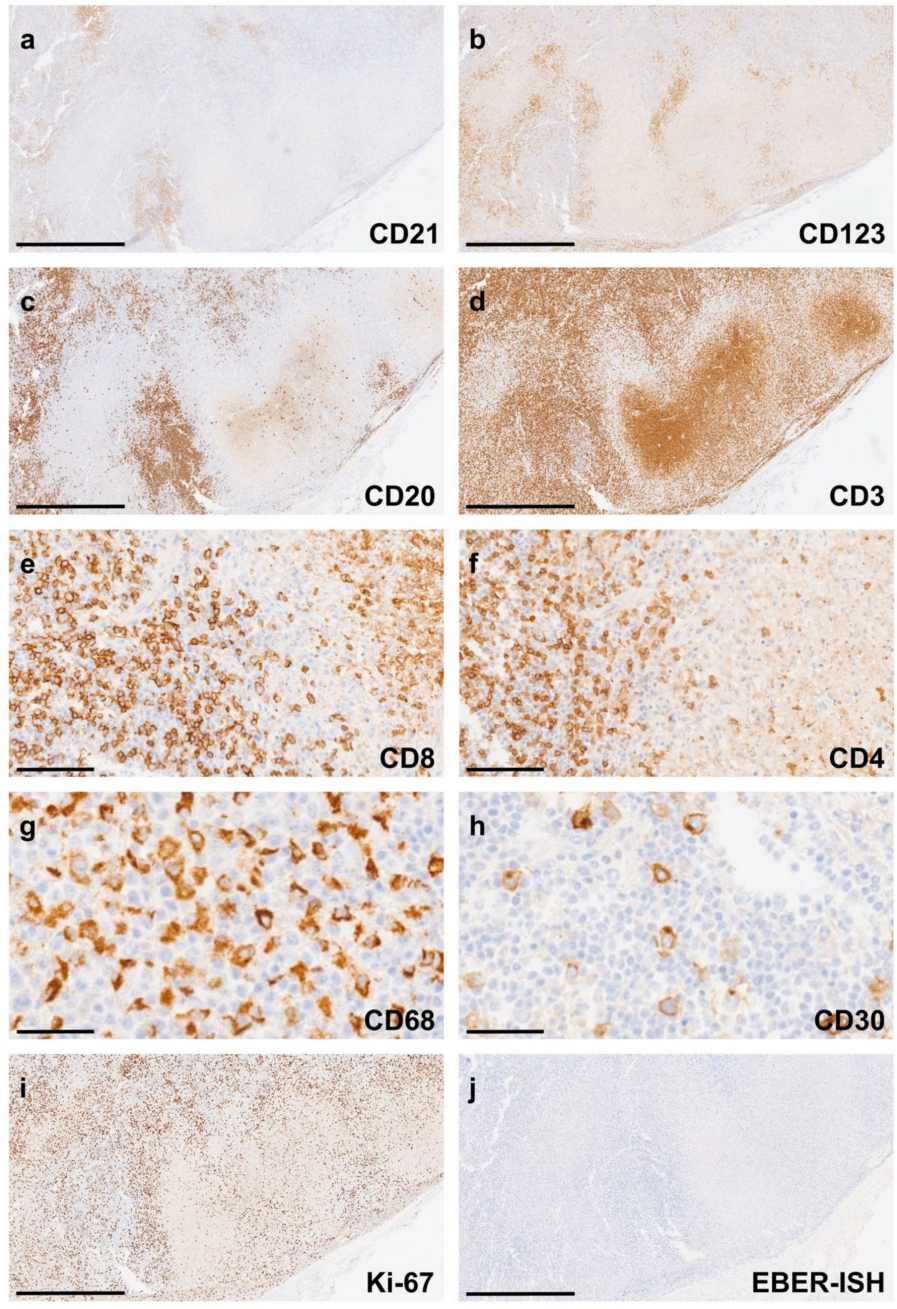
Immunohistochemical staining of the excised lymph node. **a** Disrupted CD21-positive follicular dendritic cell meshwork adjacent to necrotic areas. **b** CD123-positive plasmacytoid dendritic cells at the periphery of necrosis. **c** CD20-positive B lymphocytes near necrotic zones. **d** CD3-positive T lymphocytes. **e** CD8-positive T-cell fraction. **f** CD4-positive T-cell fraction. **g** CD68-positive macrophages within necrotic regions. **h** CD30-positive activated lymphocytes without features of Hodgkin or Reed–Sternberg cells. **i** Elevated Ki-67 expression adjacent to necrotic foci. **j** Negative Epstein–Barr virus–encoded RNA (EBER) in situ hybridization. Scale bars: **a**–**d**, **i**, **j** = 1 mm; **e**, **f** = 100 µm; **g**, **h** = 50 µm

Immunohistochemistry demonstrated a disrupted CD21-positive follicular dendritic cell meshwork adjacent to necrotic areas (Fig. 3a). Clusters of CD123-positive plasmacytoid dendritic cells were observed at the periphery of the necrosis (Fig. 3b). CD20-positive B lymphocytes were sparsely distributed at the necrotic margins (Fig. 3c), whereas CD3-positive T lymphocytes were more prominent (Fig. 3d), with a predominance of CD8-positive cells (60%) (Fig. 3e) over CD4-positive T cells (40%) (Fig. 3f). Mononuclear infiltrates within and surrounding the necrotic zones were mainly composed of CD68-positive macrophages (Fig. 3g). CD30 positivity was limited to scattered activated lymphocytes, without morphological features of Hodgkin or Reed-Sternberg cells (Fig. 3h). The proliferation index (Ki-67) was elevated adjacent to necrotic foci (Fig. 3i). In situ hybridization for Epstein-Barr virus–encoded RNA (EBER-ISH) was negative (Fig. 3j).

The immunohistochemical profile, in conjunction with the histomorphological features, confirmed the diagnosis of Kikuchi–Fujimoto disease in its necrotizing stage.

**Fig. 4:**
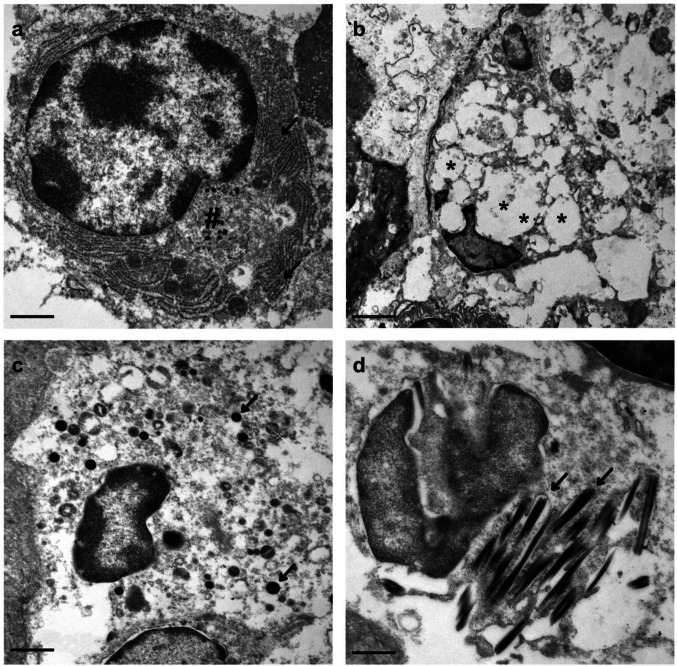
Ultrastructural findings from re-embedded paraffin material. **a** Plasma cell with rough endoplasmic reticulum (arrows) and prominent Golgi apparatus (#). **b** Probable macrophage with large cytoplasmic vacuoles (asterisks). **c** Granulocyte containing electron-dense granules (arrows). **d** Cell with unusual, rod-shaped, electron-dense inclusions (arrows) possibly located within membrane-bound compartments. No viral particles or tubuloreticular inclusions were identified. Scale bars: **a**, **c**, **d** = 1000 nm; **b** = 2500 nm

Further ultrastructural analysis of re-embedded paraffin material revealed no virus particles or definitive tubuloreticular inclusions (Fig. 4), although interpretation was limited by artifacts from the re-embedding process. Nevertheless, cells showing ultrastructural features consistent with plasma cells, macrophages, and granulocytes with preserved ultrastructural features were identified. A single cell showed unusual, electron-dense rod-shaped inclusions, possibly within membrane compartments. These resembled Auer rods, but the cell type could not be definitively classified, and no Auer bodies were observed in corresponding light microscopy.

Having established the diagnosis of KFD, the patient’s symptoms resolved completely without specific intervention during follow-up. She was managed conservatively with a “watch-and-wait” strategy with the addition of low-dose NSAID in the acute phase for symptom control.

Subsequent rheumatological evaluation by her general practitioner revealed no evidence of systemic autoimmune disease.

## Discussion

Kikuchi–Fujimoto disease (KFD) presents a significant diagnostic challenge due to its histopathologic and clinical overlap with more severe conditions, particularly lymphoma [3–5] and lupus-associated lymphadenitis [8, 11, 12, 20]. Histologically, KFD is characterized by crescent-shaped histiocytes, plasmacytoid dendritic cells, and karyorrhectic debris, notably without neutrophilic infiltration [20, 24, 25, 29]. In contrast, lupus-associated lymphadenitis frequently exhibits hematoxylin bodies, plasma cells, and fibrinoid necrosis, while lymphoma typically demonstrates clonal B or T cell proliferation, which can be confirmed via clonality analysis. The etiology of KFD remains unclear. Although infectious agents have been implicated, no definitive causal relationship has been established [6, 9, 36]. The co-occurrence of KFD with autoimmune disorders, including systemic lupus erythematodes (SLE) and Hashimoto’s thyroiditis [13, 14] as seen in this case, supports the hypothesis of an underlying autoimmune mechanism [43]. This is also supported by ultrastructural studies, where tubuloreticular bodies were found in most cases [30]. We did not find tubuloreticular inclusions or virus particles in our case, but the analysis may have been compromised by the re-embedding procedure and low sensitivity of EM. Besides supporting a link to SLE, the presence of tubuloreticular inclusions could also be interpreted as a hint for an infectious cause, as these inclusions are also found in infections with, e.g., HIV, CMV, EBV, and hepatitis B viruses [44]. Interestingly, we found a cell with peculiar rod-like inclusions, showing similarities with Auer bodies. While Auer bodies are typical for leukemic cells [45], we excluded a neoplastic process in our case and also did not find Auer bodies in light microscopy. Interpretation of these structures thus currently remains speculative, and their nature could be further elucidated in future studies. While analysis of re-embedded material for EM can provide valuable information in, e.g., emerging infectious diseases such as COVID-19 and mpox infection [46, 47], special care needs to be taken to avoid overinterpretation of findings, and direct processing of samples for EM should be prioritized. Genetic studies have identified specific HLA alleles associated with increased susceptibility [31, 48], suggesting that certain populations may have a predisposition to developing KFD. KFD is generally a self-limiting condition, with symptoms resolving within weeks to months [20, 23, 37, 38]. In the present case, symptoms resolved gradually over the course of several weeks without specific interventions. While corticosteroids may be considered in severe or persistent cases, most patients can be managed conservatively using a “watch-and-wait” approach [9, 10, 34–36]. Recognizing KFD is critical to avoid unnecessary and potentially harmful interventions such as chemotherapy or immunosuppressive therapy, which may be initiated if the condition is misdiagnosed as lymphoma. Although KFD is a benign disorder, recurrences can occur months after initial resolution [42]. Additionally, several autoimmune diseases have been reported to develop in patients following an episode of KFD [23]. These observations highlight the importance of early and accurate diagnosis, as well as long-term follow-up in patients presenting with cervical lymphadenopathy [23, 49].

## Conclusion

Kikuchi–Fujimoto disease is a rare but clinically relevant differential diagnosis in young patients presenting with cervical lymphadenopathy. Its characteristic histological and immunohistochemical features are key to distinguishing it from lymphoma and autoimmune lymphadenitis. As KFD is typically self-limiting, early recognition is essential to prevent unnecessary and potentially harmful treatments. Given its potential association with autoimmune disease and recurrence, long-term clinical monitoring is advisable. Further studies are warranted to elucidate its pathogenesis and identify specific diagnostic markers.

## Data Availability

All data presented is available in anonymized form on request.
